# Castration promotes the browning of the prostate tumor microenvironment

**DOI:** 10.1186/s12964-023-01294-y

**Published:** 2023-09-28

**Authors:** Alejandro Alvarez-Artime, Belen Garcia-Soler, Pedro Gonzalez-Menendez, Sheila Fernandez-Vega, Rafael Cernuda-Cernuda, David Hevia, Juan C. Mayo, Rosa M. Sainz

**Affiliations:** 1 Departamento de Morfologia y Biologia Celular, Facultad de Medicina, University of Oviedo, Julian Claveria 6, 33006 Oviedo, Spain; 2 Instituto Universitario de Oncología del Principado de Asturias (IUOPA), 33006 Oviedo, Spain; 3 Instituto de Investigación Sanitaria del Principado de Asturias (ISPA), Avda. Hospital Universitario, 33011 Oviedo, Spain

**Keywords:** Prostate cancer, Browning, Adipose tissue, Tumor microenvironment, UCP1

## Abstract

**Background:**

Adipose tissue has gained attention due to its potential paracrine role. Periprostatic adipose tissue surrounds the prostate and the prostatic urethra, and it is an essential player in prostate cancer progression. Since obesity is directly related to human tumor progression, and adipose tissue depots are one of the significant components of the tumor microenvironment, the molecular mediators of the communication between adipocytes and epithelial cells are in the spotlight. Although periprostatic white adipose tissue contributes to prostate cancer progression, brown adipose tissue (BAT), which has beneficial effects in metabolic pathologies, has been scarcely investigated concerning cancer progression. Given that adipose tissue is a target of androgen signaling, the actual role of androgen removal on the periprostatic adipose tissue was the aim of this work.

**Methods:**

Surgical castration of the transgenic adenocarcinoma of the mouse prostate (TRAMP) was employed. By histology examination and software analysis, WAT and BAT tissue was quantified. 3T3-like adipocytes were used to study the role of Casodex® in modifying adipocyte differentiation and to investigate the function of the secretome of adipocytes on the proliferation of androgen-dependent and independent prostate cancer cells. Finally, the role of cell communication was assayed by TRAMP-C1 xenograft implanted in the presence of 3T3-like adipocytes.

**Results:**

Androgen removal increases brown/beige adipose tissue in the fat immediately surrounding the prostate glands of TRAMP mice, concomitant with an adjustment of the metabolism. Castration increases body temperature, respiratory exchange rate, and energy expenditure. Also, in vitro, it is described that blocking androgen signaling by Casodex® increases the uncoupling protein 1 (UCP1) marker in 3T3-like adipocytes. Finally, the effect of brown/beige adipocyte secretome was studied on the proliferation of prostate cancer cells in vivo and in vitro. The secretome of brown/beige adipocytes reduces the proliferation of prostate cancer cells mediated partly by the secretion of extracellular vesicles.

**Conclusions:**

Consequently, we concluded that hampering androgen signaling plays a crucial role in the browning of the periprostatic adipose tissue. Also, the presence of brown adipocytes exhibits the opposite effect to that of white adipocytes in vitro regulating processes that govern the mechanisms of cell proliferation of prostate cancer cells. And finally, promoting the browning of adipose tissue in the periprostatic adipose tissue might be a way to handle prostate cancer cell progression.

Video Abstract

**Supplementary Information:**

The online version contains supplementary material available at 10.1186/s12964-023-01294-y.

## Background

Prostate cancer (PCa) is the most frequently diagnosed tumor among men in Western countries [1]. Despite its high incidence, prostate tumors grow slowly and remain confined to the gland. However, some tumors overgrow, develop chemo and hormone resistance, and acquire a highly invasive and metastatic phenotype [2]. The evolution of the prostate tumors is still unpredictable. However, there are no prognostic markers. The prostate gland is highly dependent on androgens for its normal development and function [3]. In PCa patients, anti-hormonal therapy causes an initial reduction of the tumor [4]. Nevertheless, in some patients, this tumor develops a hormone-refractory phenotype, resistant to anti-hormonal treatment, that is associated with a poor prognosis [5].

In PCa, the role of the tumor microenvironment is crucial during tumor growth and progression. The periprostatic adipose tissue (PPAT), which surrounds seminal vesicles and the prostate, has been reported to contribute actively to tumor growth and progression, mainly through paracrine mechanisms [6]. Classically, two main types of adipose tissue have been described, i.e. white adipose tissue (WAT) with an energy storage function, and brown adipose tissue (BAT) with a thermogenic activity Mitochondrial BAT are characterized by the presence of thermogenin, called uncuoupling protein 1 or UCP-1 that generates non-shivering thermogenesis [7]. Excess of WAT is commonly clear evidence of the overweight phenotype, characterized by an energy imbalance generally caused by an increased caloric intake and a decreased energy expenditure (EE). In fact, a metabolic switch in adipocytes promotes accumulation of triglycerides, activation of lipogenic pathways, and alterations in the secretory profile [8]. While it has been clearly demonstrated that WAT is involved in cancer progression [9], BAT has gained a high scientific interest in the last decade because of its clinical relevance in metabolic and cardiovascular diseases [10, 11]. Regarding this, BAT activation by cold exposure or pharmacological interventions such the antidiabetic rosiglitazone (RSGZ) or thermogenic agent like β-adrenergic agents elicits beneficial effects through the triggering of lipolysis and fatty acid β oxidation, which ultimately increases energy expenditure and insulin sensitivity, showing the opposite role in the metabolism to that of WAT [12].

In 1981 Young et al*.* described the so-called WAT ‘browning’, a process triggered by the expression and activation of specific signaling pathways [13]. This high plasticity of adipose tissue because of its easy differentiation, dedifferentiation, or transdifferentiation potential is another way to reduce WAT content. It has been recently found that browning has beneficial activities in adipose tissue-associated pathologies [14]. In cancer, a valuable role of brown/beige adipose tissue was recently described by causing tumor suppression activity through the cold-altered global metabolism [15]. Nevertheless, the role of brown/beige adipose tissue in PCa remains unclear.

WAT is a well-known risk factor for PCa. However, the role of androgens and androgen receptor (AR) in the browning of PPAT, as well as the impact of this brown/beige phenotype in PCa growth and progression is still poorly understood. Therefore, the main objective of this work was to study the role of androgens in the adipogenic differentiation of brown/beige PPAT and its role in prostate tumor progression.

## Material and methods

### Reagents

All reagents, equipment, companies, and references used in this article are shown in Supplementary Table 1.

### Cell lines

Murine embryonic fibroblast 3T3-L1 cells (ATCC, #CL-173™) were maintained in a proliferative medium (PM) made up of DMEM high glucose (4.5 g/L glucose), supplemented with 10% newborn calf serum, 4 mM L-glutamine, 1 mM, sodium pyruvate and 1% antibiotic–antimycotic cocktail (100 U/mL penicillin, 10 µg/mL streptomycin, 0.25 µg/mL amphotericin B). Murine PCa cells TRAMP-C1 (ATCC, #CRL-2730™) were cultured in DMEM high glucose supplemented with 5% fetal bovine serum (FBS), 4 mM L-glutamine, 5% Nu-serum IV complement, 0.005 mg/mL insulin, 10 nM dehydroepiandrosterone (DHEA) and 1% of an antibiotic–antimycotic cocktail. Human androgen-dependent LNCaP cells (ATCC, #CRL-1740) were cultured in RPMI 1640 supplemented with 10% FBS, 2 mM L-glutamine, 15 mM HEPES, and 1% of antibiotic cocktail (100 U/mL penicillin, 10 µg/mL streptomycin). Human androgen-independent PC-3 cells (ATCC, #CRL-1435) were cultured in DMEM/F12 medium supplemented with 10% FBS, 2 mM L-glutamine, and 1% of an antibiotic–antimycotic cocktail. All cell lines were cultured at standard conditions (T^ª^: 37 oC, CO_2_: 5%). FBS was stripped from small weight molecules, including steroids, by using a protocol previously published [16]. Charcoal-stripped FBS (csFBS) was employed to perform a cell culture medium without androgens as a substitute of FBS.

### Animal model and procedures

Transgenic adenocarcinoma of the mouse prostate [C57BL/6-Tg (TRAMP)8247Ng] mice were purchased from Jackson Laboratories. Mice were bred and maintained in our animal-building facilities. All animals were exposed to a 12:12 light–dark cycle with food and water ad libitum at 22 oC and 50% humidity. Three weeks after birth, experimental animals were genotyped using the primers described in Supplementary Table 2. All the experiments were designed with the approval of the Ethics Committee in Animal Experimentation of the University of Oviedo. Surgical procedures were carried out following the European Directive 2012/63/EU (PROAE 32/2016, PROAE 01/2020, PROAE 20/2021).

Bilateral male orchiectomy was performed at 12 or 20 weeks under anesthesia by i.p. injection with 100 mg/kg of ketamine and 20 mg/kg of xylazine. A small incision of 1–2 cm was performed in the peritoneum and the testis and epididymal fat (eWAT) were excised by cauterization. In the sham-operated animals only incisions and sutures were performed. For exogenous testosterone (TEST) administration, 2.5 mg/kg of body weight of TEST was s.c. daily administrated. All animals were euthanized after 4 weeks of treatment.

For open circuit indirect calorimetry, WT and TRAMP animals (sham, CAST and CAST + TEST) were analyzed by respirometry in an OXYMAX system (Columbus, OH, USA). Animals were placed in individual cages and monitored for 48 h. Oxygen zonconsumption and CO_2_ production were registered every 10 min. Data from the first 24 h was discarded considering this a period of acclimatization.

To induce the browning of white adipose tissue, 16 weeks old WT, and TRAMP animals were treated with the PPAR-ɣ activator rosiglitazone (RSGZ). The drug was dissolved in DMSO and daily administered at 10 mg/kg bw for 10 days, following a protocol previously published by Ohno et al. in 2012 [17]. For long-term administration, RSGZ was injected every 72 h until animals were 24 weeks old. During the experiment, body weight was registered weekly and after euthanasia GU tract, eWAT, subcutaneous WAT (scWAT), and interscapular BAT (iBAT) were collected, weighed, and frozen in liquid nitrogen.

### Differentiation of 3T3-L1 cells to adipocytes

3T3-L1 cells were cultured in differentiation medium (DM): DMEM/F12 supplemented with 10% FBS, 4 mM L-glutamine, 1 mM sodium pyruvate and 1% of an antibiotic–antimycotic cocktail. 3T3-L1 cells were seeded at a density of 5.000 cells/cm^2^ in PM. At confluency DM was added and supplemented with a differentiation cocktail: 1 μM dexamethasone, 0.5 mM 3-isobutyl-1-methylxanthine (IBMX), 2 μM RSGZ and 10 μg/mL of human insulin. Cells were maintained during 48 h in DM. After 48 h DM was removed, and fresh DM only with 10 μg/mL insulin was added. White adipogenesis differentiation was concluded 10 days after the induction of the process.

### Organotypic culture and differentiation of adipose tissue

For the differentiation of WAT into brown/beige adipose tissue, we first excised a small piece of eWAT from a donor mouse. Fat pads were washed in phosphate buffered saline (PBS) pH 7.4 and gently cut into small pieces (2–5 mm). 5–6 adipose tissue explants were distributed in 60 mm well plates and cultured for 24 h in DMEM high glucose supplemented with 10% FBS, 4 mM L-glutamine, 1 mM sodium pyruvate, 20 mM HEPES, 50 µg/mL sodium ascorbate, 1 µM insulin and 1% of antibiotic–antimycotic cocktail (100 U/mL penicillin, 10 µg/mL streptomycin). After 24 h of culture, differentiation procedure was started by the addition to the culture medium of a differentiation cocktail modified from Blumenfield et al*.* [18]: 1 µM dexamethasone, 0.5 mM IBMX, 50 µM indomethacin, 2 µM RSGZ, 250 nM triiodothyronine. DMSO was added to control tissue. Adipose tissue fragments were cultured for 3 weeks. Subsequently, explants were recovered, and differentiation was confirmed by *UCP1* expression using the primers indicated in Supplementary Table 2*.*

### Oil red O staining

For the staining of neutral lipids, cells were fixed in a solution of 4% paraformaldehyde (PFA) in 0.1 M phosphate buffer (PB) pH 7.4 for 30 min at 4 oC. Cells were permeabilized with 60% of 2-propanol for 2 min at room temperature (RT) and stained with a 3:2 Oil Red O (ORO) solution of 3 mg/mL diluted in distilled water for 10 min. Nuclei were stained with hematoxylin for 10 min. ORO was extracted by the addition of 100% of 2-propanol to the cells with gentle agitation for 5 min at RT and absorbance was analyzed at 520 nm in an Biotek Sinergy H1 microplate reader.

### 5-ethynyl-2-deoxyuridine (EdU) labeling of cells and prostate tumor tissue

TRAMP-C1 cells were seeded at a density of 5,000/cm^2^ cells on polycarbonate Transwell™ inserts with a 0.4 µm mesh. All samples were further stained with 5-ethynyl-2-deoxyuridine (EdU), according to the manufacturer protocol (Baseclick GmbH, Neuried, Munich, Germany). Slides containing tissue sections were then deparaffined, hydrated and mounted with Fluoromount™ mounting medium, while for the observation of TRAMP-C1 cells, Transwell™ inserts were disassembled from the scaffold, placed on a slide, and mounted. Samples were observed in an epifluorescence microscope Nikon Eclipse 80i using FITC (λ_ex_ 465–495 nm; λ_em_ 515–555 nm; DM505) for EdU, or DAPI filters (λ_ex_ 340–380 nm; λ_em_ 435–485 nm, DM400). For the quantification of EdU, images were taken at 100 × magnification, and the ratio between EdU-positive cells and total cells stained with DAPI was calculated.

### Extracellular vesicles isolation

For the isolation of extracellular vesicles (EVs) secreted by 3T3-L1 cells, 3T3-L1^naive^ or differentiated cells were seeded in 150 mm plates in DM with 10% of free-EVs-FBS and 10 μg/mL of insulin. EVs were isolated by differential ultracentrifugation at 120.000 xg for 70 min. Sedimentation efficiency was assayed by DLS, and protein concentration was quantified by bicinchoninic acid assay (BCA). EVs were frozen at -80 °C until use.

### RNA isolation and qPCR analysis

RNA isolation was performed by a phenol–chloroform extraction using TRI Reagent. (Sigma Aldrich, Merck, Massachusetts, MA, USA). Samples were run in triplicates and qPCR was carried out by using SYBER Green Master Mix, in an Applied Biosystems QuantStudio 5 qPCR system. Sequence-specific primers are shown in Supplementary Table 2.

### Chromatin immunoprecipitation (ChIP)

DNA was extracted using a kit provided by Cells Signaling Technology. 15 µg of DNA were incubated with 4 µg of anti-AR antibody (Supplementary Table 3) overnight with gentle shaking at 4 oC. Samples were incubated with magnetic A/G protein beads, AR was detached, and the sequence was purified according to the manufacturer’s instructions. To confirm the union of AR to the *Ucp1* promoter DNA sequence was amplified and the PCR product was sequenced. Alignments were performed using MEGA 11 software and the algorithm MUSCLE.

### Morphometric and morphological analysis

Genitourinary (GU) tracts were fixed with 4% PFA in 0.1 M PB, pH 7.4, overnight. Subsequently, they were embedded in paraffin and 5-µm sections were obtained. These sections were deparaffined, hydrated, and stained with hematoxylin–eosin. Next, they were dehydrated, cleared in eucalyptol, and mounted in a hydrophobic medium (Eukitt). For the PPAT analysis, white adipocytes average area, white/beige adipose tissue area, and intratumor white adipocytes were measured. Ten images were taken at 100 × magnification, and the surface was calculated using ImageJ/Fiji software. For analysis of the white/beige area, slides were scanned in a Leica SCN 400 at 1 × magnification, and adipose tissue and prostate gland area were measured.

### Toluidine blue staining

5-µm paraffin-embedded tissue sections were obtained, deparaffined, hydrated, and stained for 2 min with 0.1% toluidine blue solution at RT, dehydrated and mounted in Eukitt®. Five representative images were taken at an initial magnification of 200 × and metachromatic cells per gland surface were quantified.

### Immunohistochemistry

For the UCP1 detection slides were placed in a 0.1 M of TRIS–HCl pH 7.5 buffer at 37 °C 30 min, and samples were incubated with a 0.05% pronase solution in 0.1 M of TRIS–HCl pH 7.5. For anti-AR, anti-KI67, anti-CD11c, anti-CD86, anti-C206 or anti-Arginase 1 immunolabeling, antigen retrieval was performed in all cases by incubation at 95 °C in 10 mM sodium citrate buffer pH 6.0 for 25 min. In the case of anti-F4/80, no antigen retrieval was required. Samples were blocked with a solution of goat serum for 2 h at RT and reacted with the primary antibodies described in Supplementary Table 3. Samples were then incubated with FITC-conjugated secondary antibodies, counterstained with 1 µg/mL of DAPI and mounted with Fluoromount. Samples were observed in a Nikon Eclipse 80i epifluorescence microscope using specific filters for FITC immunostaining (λ_ex_ 465–495 nm; λ_em_ 515–555 nm; DM505) and for DAPI counterstaining (λ_ex_ 340–380 nm; λ_em_ 435–485 nm, DM400).

### Statistical analysis

Data are presented as the mean ± standard error of the mean (SEM), or by the area under the curve (AUC). Under a normal distribution, statistical differences were assayed by one-way ANOVA, followed by post-hoc Student–Newman–Keuls multiple t-tests. Under no normal distribution, data were analyzed by ANOVA on ranks with a Kruskal–Wallis post-hoc. Statistical differences were considered (and represented in the graphs with the asterisk in parenthesis) when p < 0.05 (*), *p* < 0.01 (**), *p* < 0.001 (***), and *p* < 0.0001 (****).

## Results

### Castration induces brown/beige adipose tissue in the prostate tumor microenvironment

At the histological level, the prostate gland of WT mice has a cubic simple epithelium, with central rounded nuclei and loose and sparsely cellular connective tissue. By contrast, castrated mice have a denser and cellular stroma with basal and rounded nuclei. In TRAMP mice, an increase in the number of small glands with a higher cell density and prismatic morphology was found. Epithelial cells have basally located hyperchromatic nuclei and, in some cases, show an elongated morphology, which are the typical features of tumor tissue. An increase in cellular and matrix density was also found in the stroma. Nevertheless, castration promoted the recovery of the non-tumor phenotype, showing atrophic glands with the histological features of WT mice. Moreover, the stroma adjacent to the glands was dense, but became looser in remote regions (Supplementary Fig. 1A). As expected, castration in WT mice did not affect the GU/ body weight (bw) ratio while in TRAMP mice the GU *vs* bw ratio was significantly reduced after 4 or 12 weeks of castration (Supplementary Fig. 1B).

Furthermore, this reduction was confirmed by the analysis of prostate cell proliferation by EdU labeling, which showed a high rate of proliferation among the epithelial cells in TRAMP mice and a significant reduction after castration (Supplementary Fig. 1C).

**Fig. 1:**
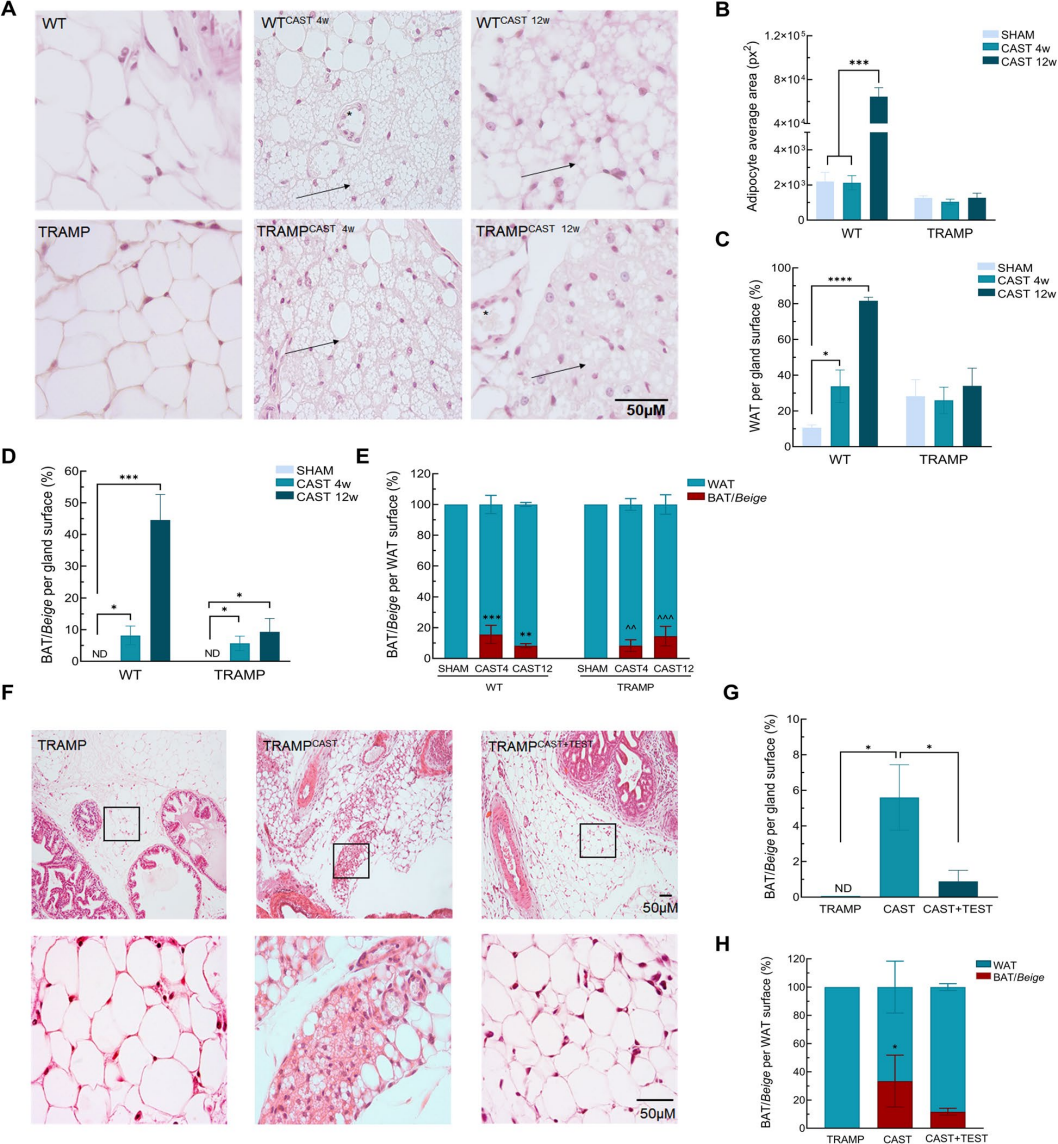
Castration induces Brown/beige adipose tissue in the prostate tumor microenvironment. **A** Effect of castration on the PPAT. Representative images of hematoxylin–eosin slides from WT and TRAMP mice after castration for 4 (CAST 4w) or 12 (CAST 12w). Arrow shows multilocular adipocytes depots. Images were taken at 400X of magnification. **B** Average size of white adipocytes. **C** WAT surface per total gland surface. **D** BAT/beige fat surface per total gland surface. **E** Brown/beige fat surface vs WAT surface ratio in SHAM operated and after for 4 (CAST 4w) or 12 (CAST 12w) of castration. **F** effect of 2.5 mg/Kg testosterone administration for 5 days in the PPAT.of TRAMP, TRAMP castrated (CAST), and TRAMP castrated plus testosterone (CAST + TEST). Arrow shows brown/beige fat depots. (*) Blood vessel. Images are taken at 100 and 400X of magnification. **G** BAT/beige fat surface per total gland surface after castration and testosterone administration. **H** Brown/beige fat surface vs WAT surface ratio after castration and testosterone administration. Figures A-E: *n* = 10. Figures F-G: *n* = 5. ND: non detectable. Data are presented as mean ± SEM. * *p* < 0.05, **p < 0.01, *** *p* < 0.001, **** *p* < 0.0001

Along with the prostate atrophy, morphological changes of PPAT were found in castrated mice. Brown/beige multilocular adipocytes were frequently observed within the white PPAT after castration (Fig. 1A). Morphometric analysis showed that 12-week castrated-induced PPAT from WT mice contained bigger white adipocytes than that of sham-operated WT mice. In TRAMP mice, castration did not show differences in white adipocyte size (Fig. 1B). In addition, an increase in the WAT surface was found in either 4- or 12-week castrated WT animals, whereas TRAMP mice did not show differences after castration (Fig. 1C). However, a significant rise in brown/beige fat was found in both genotypes after castration (Fig. 1D). Finally, the brown/beige *vs* WAT ratio measured on both genotypes showed a significant increase both after 4- and 12-weeks post-orchiectomy (Fig. 1E).

To confirm that the previous results were directly related to androgen signaling, testosterone was administered to TRAMP mice. Daily s.c. administration of testosterone recovered the growth of the prostate, reflected by GU *vs* body weight ratio and gland morphology examination (Supplementary Fig. 1D). Testosterone administration partially prevented castration-induced increased brown/beige fat depots within the white PPAT (Fig. 1F), as also confirmed by morphometric analysis (Fig. 1G, H).

The presence of adipose tissue within the prostate tumor was obvious at microscopic level, confirming the occurrence of intratumor white adipocytes along with the prostate tumor progression. Thus, isolated white adipocytes were only detected in the periphery of poorly differentiated (PD) tumors but were scarce in well (WD) or moderately differentiated (MD) tumors (Supplementary Fig. 2).

### Castration increases energy expenditure in TRAMP mice

**Fig. 2:**
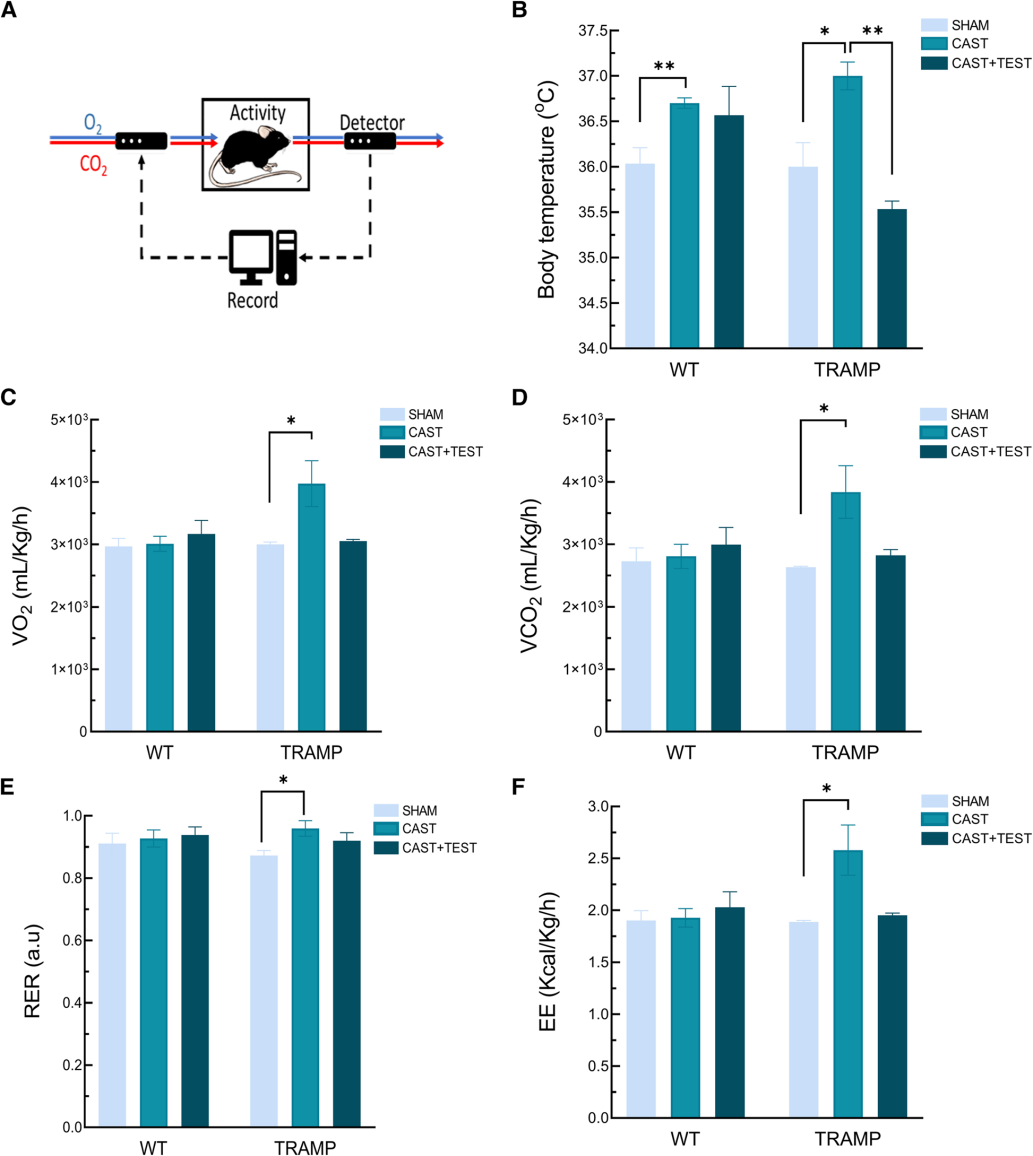
Castration increases energy metabolism in TRAMP mice. **A** Representation of Oxymax open-circuit indirect calorimetry procedure. **B** Body temperature of WT and TRAMP mice after castration (CAST) and 2.5 mg/Kg of testosterone administration (CAST + TEST). **C** Mice oxygen consumption (VO_2_) (**D**) Carbon dioxide production (VCO_2_) (E) Respiratory exchange ratio (RER), (**F**) Energy expenditure (EE). *n* = 3. Data are represented as area under the curve (AUC) ± SEM. *p < 0.05, ** *p* < 0.01

Since castration increases the brown/beige depots in the PPAT, and this fat has an important thermogenic activity, rectal temperature was measured. Also, adipose tissue has an important role in metabolic homeostasis and therefore, metabolic profiles of either, sham or castrated TRAMP mice, were analyzed by using open-circuit indirect calorimetry, as well as after the restitution of androgens with exogenous testosterone administration (Fig. 2A). Oxygen consumption (VO_2_) and carbon dioxide production were measured (VCO_2_) during a 24 h cycle and the respiratory exchange ratio (RER) and energy expenditure (EE) were calculated.

As expected, in both genotype, WT and TRAMP, castration promoted a significant increase in body temperature. Additionally, in TRAMP castrated mice, testosterone administration prevented the increase in body temperature, getting similar values to sham-operated animals. Testosterone treatment showed no effect in rectal temperature in WT mice (Fig. 2B). Finally, TRAMP mice consumed more O_2_ and produced more CO_2_ after castration. This rise was also reflected in the increase of RER and EE. In these transgenic animals, testosterone recovered all parameters to the control levels of sham-operated TRAMP animals. Again, in WT no differences were observed in the core metabolism between groups, with similar values in VO_2_ and VCO_2_. (Fig. 2C-F).

### Castration increases UCP1 levels and changes AR location in PPAT

**Fig. 3:**
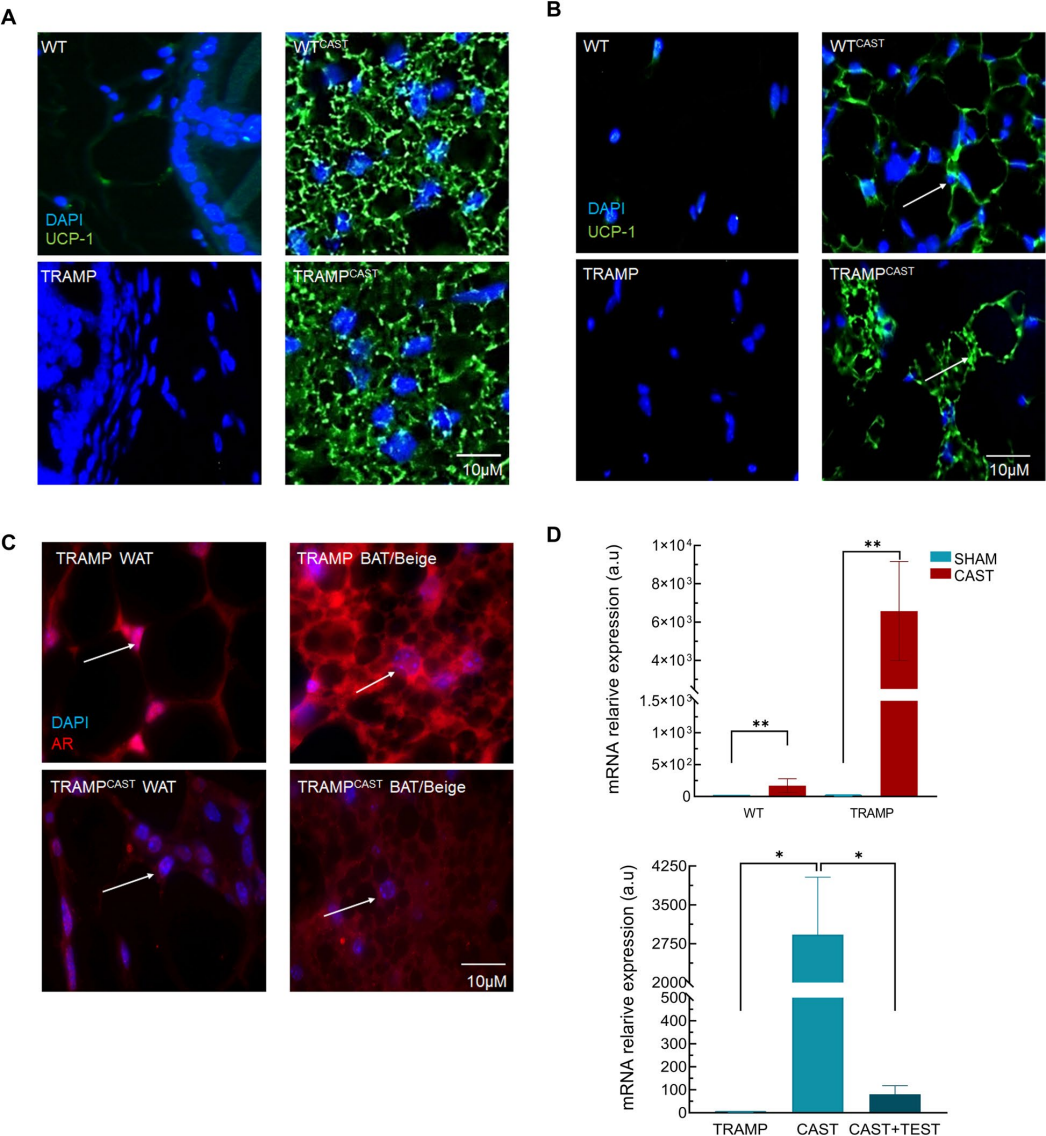
Castration increases UCP1 levels and changes AR location in the PPAT. **A** UCP1 location in the PPAT of SHAM operated WT and TRAMP mice and after castration (CAST). Arrows shows positive immunolabeling in the brown/beige adipocytes. Images were taken at 40 and 400X of magnification. **B** UCP1 location in the white PPAT. Arrows shows positive immunolabeling for UCP1 in white adipocytes. Images were taken at 40 and 400X of magnification. **C** AR location in the PPAT of TRAMP and TRAMP castrated (CAST) mice. Arrow shows subcellular location of AR. Images were taken at 100 and 400X of magnification. **D** Upper panel: expression level of Ucp1 in WT and TRAMP animals after 12 weeks of castration (CAST) *n* = 6. Lower panel: expression levels of Ucp1 in TRAMP mice after 4 weeks of castration (CAST) and before 2.5 mg/Kg of testosterone administration (CAST + TEST) *n* = 5. Data are presented as mean ± SEM. * *p* < 0.05, ** *p* < 0.01

Since castration increase the presence of brown/beige adipose tissue in the PPAT, production of BAT maker UCP1 was analyzed. Positive immunolabeling for UCP1 was detected in the adipose tissue surrounding the glands of castrated mice from both genotypes. This labeling identified multilocular depots and was found mainly in BAT adipocytes (Fig. 3A). However, it is noteworthy to mention that in some areas of the PPAT of castrated animals, UCP1 was detected in the WAT adipocytes located in the vicinity of the brown/beige tissue, suggesting a process of transdifferentiation (Fig. 3B).

AR location was studied in the prostate and PPAT of TRAMP mice after castration. Glandular epithelium of TRAMP mice showed a clear nuclear AR location in either well (WD) and moderately (MD) differentiated tumors. However, AR location in castrated mice was mostly cytoplasmic, and in some cases even undetectable (Supplementary Fig. 3). In the PPAT, AR location changed between WAT and brown/beige depots. Thus, in WAT adipocytes, AR was located mainly in the nucleus, while in brown/beige depots a cytoplasmic location was found (Fig. 3C).

Moreover, *Ucp1* expression was analyzed in WT and TRAMP mice after castration. *Ucp1* expression in eWAT significantly increased in both, WT and TRAMP animals, after 12-week castration. Moreover, exogenous testosterone significantly reduced *Ucp1* expression in TRAMP mice 4 weeks after castration, confirming a potential role for androgens on browning of adipose tissue (Fig. 3D).

### RSGZ treatment increased iBAT but did not reduce prostate tumor progression

**Fig. 4:**
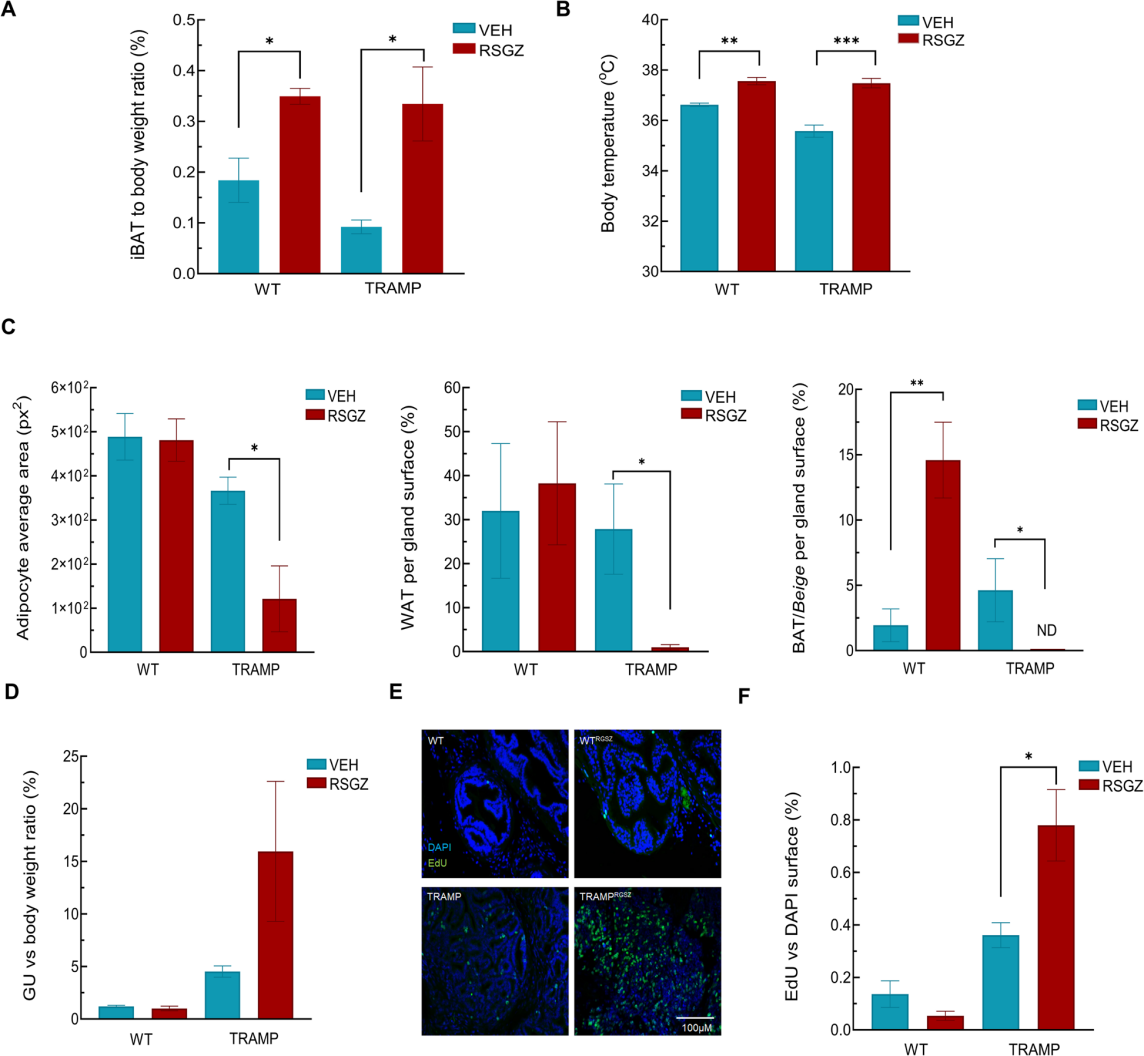
Effect of RSGZ treatment for 6 weeks in the prostate tumor microenvironment. **A** iBAT weight vs body weight ratio in WT and TRAMP mice after RSGZ administration. **B** Body temperature in WT and TRAMP after 6 weeks of RSGZ treatment. **C** Morphometric analysis of PPAT: adipocyte average area (left panel), WAT surface vs gland (central panel), brown/beige fat surface vs gland (right panel) (**D**) Effect of RSGZ treatment on the GU vs body weight ratio (right panel) (**E**) Effect of RSGZ treatment for 6 weeks in the prostate epithelial cells proliferation by EdU Images were taken at 400X of magnification. **F** Number of positive cells for EdU vs DAPI surface 5 different images form each mouse were used for the quantification. *n* = 5. Data were represented as mean ± SEM. * *p* < 0.05, ** *p* < 0.01, **** *p* < 0.0001

To study whether increment of brown/beige depots affect prostate tumor progression in the presence of circulating androgens, a pharmacologic strategy of browning fat was employed. For this purpose, rosiglitazone (RSGZ) was chronically injected in WT and TRAMP mice to induced browning in the adipose tissue. As internal control for RSGZ-induced browning, interscapular BAT (iBAT) was collected and weighted. RSGZ treatment significantly increased the iBAT in both genotypes (Fig. 4A), concomitant with an increase in body temperature (Fig. 4B). However, no differences were found in other energetic/metabolic parameters (VO_2_, VCO_2_, RER, EE; data not shown). After RSGZ treatment, TRAMP PPAT showed a dramatic reduction in WAT content and in the size of white adipocytes. However, while RSGZ promoted a significant increase in BAT in WT animals, RSGZ-treated TRAMP mice displayed no brown/beige depots within the PPAT (Fig. 4C). More importantly, not only did RSGZ not altered GU/bw ratio but also, it increased cell proliferation in TRAMP mice (Fig. 4D). Even more, RSGZ-treated mice displayed more aggressive, poorly differentiated (PD) tumors (4 out of 5) (Fig. 4E, F).

### Castration modulates the number of macrophages and mast cells in the microenvironment of the prostate tumor

**Fig. 5:**
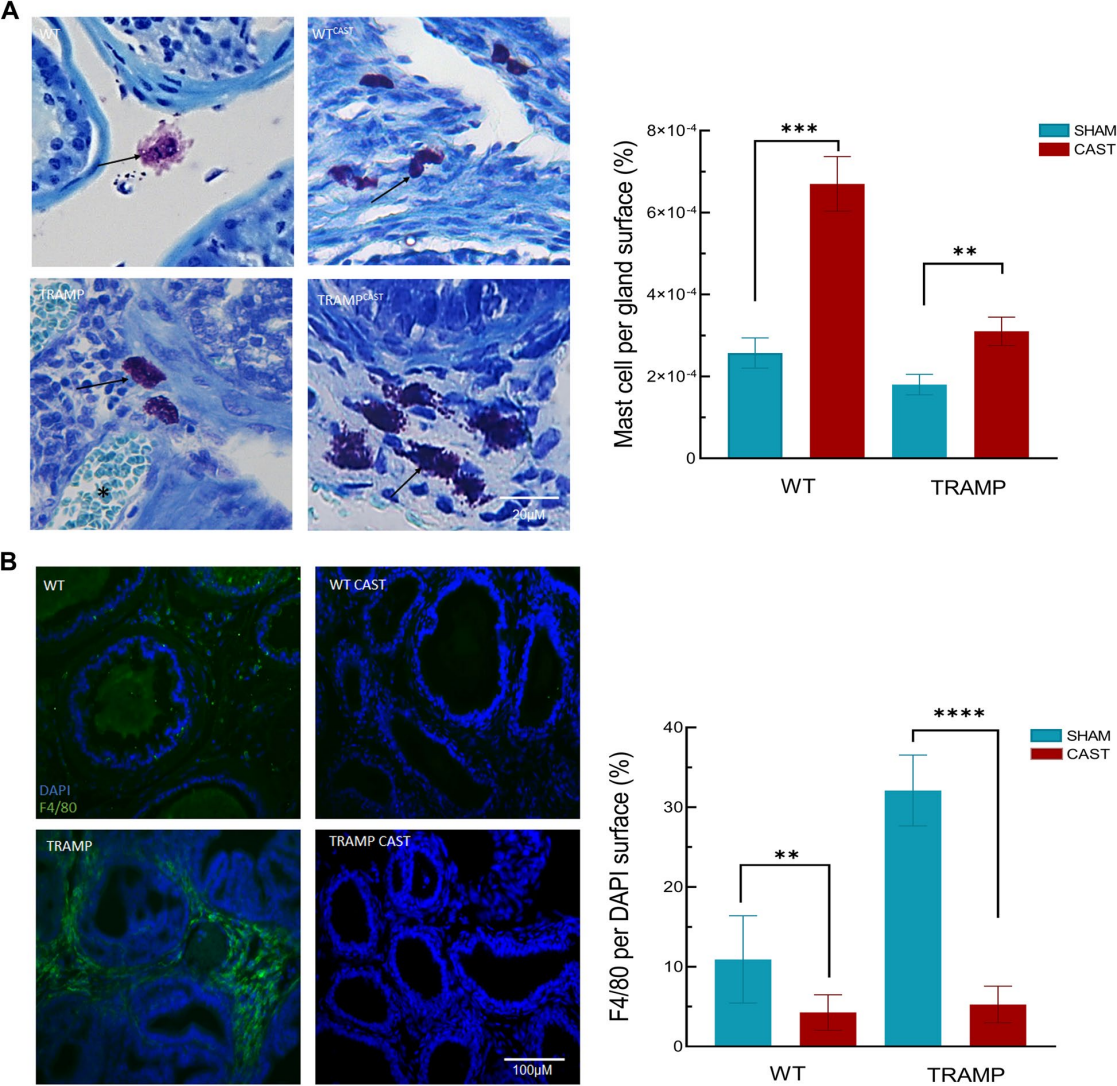
Castration induces PPAT browning, reduces macrophage infiltration. **A** Toluidine blue stain of slides from 24 weeks old WT and TRAMP mice after 12 weeks of castration. Arrows shows metachromatic cells (mast cell) in the prostate stroma (left panel). Images were taken at 400X of magnification. Mast cell quantification per total gland surface (right panel). **B** Macrophages detection in the prostate stroma from 24 weeks old WT and TRAMP mice after 12 weeks of castration (left panel). Arrows shows F4/80 positive cells. Quantification of F4/80 surface per DAPI surface (right panel). Arrows shows F4/80 positive cells. Quantification of F4/80 surface per DAPI surface (right panel). For all quantifications, 5 different images from each mouse were analyzed. *n* = 5. Data are presented as mean ± SEM. * *p* < 0.05, ** *p* < 0.01, *** *p* < 0.001, **** *p* < 0.0001

Since a reduction of WAT and its transdifferentiation into brown/beige fat could be related to the inflammatory status of the prostate microenvironment, both mast cell and macrophages were studied 12 weeks after castration. Castration significantly increased the number of mast cells among the glands in both WT and TRAMP mice (Fig. 5A). The number of macrophages within this prostatic stroma was much higher in TRAMP compared to WT mice and this increase was totally prevented by castration (Fig. 5B). Macrophage polarization in the M1/M2 phenotypes was analyzed and we found that both macrophage subpopulations were equally reduced in number by castration (Supplementary Fig. 4).

### Androgens negatively regulate UCP1 expression in adipocyte-like 3T3-L1 cells

Since absence of androgen seems to be essential for the occurrence of BAT depots within the PPAT, we tried to elucidate the role of androgen signaling on browning by using the murine embryonic fibroblast 3T3-L1 cell line, which possesses a high potential for differentiation into adipocytes.

**Fig. 6:**
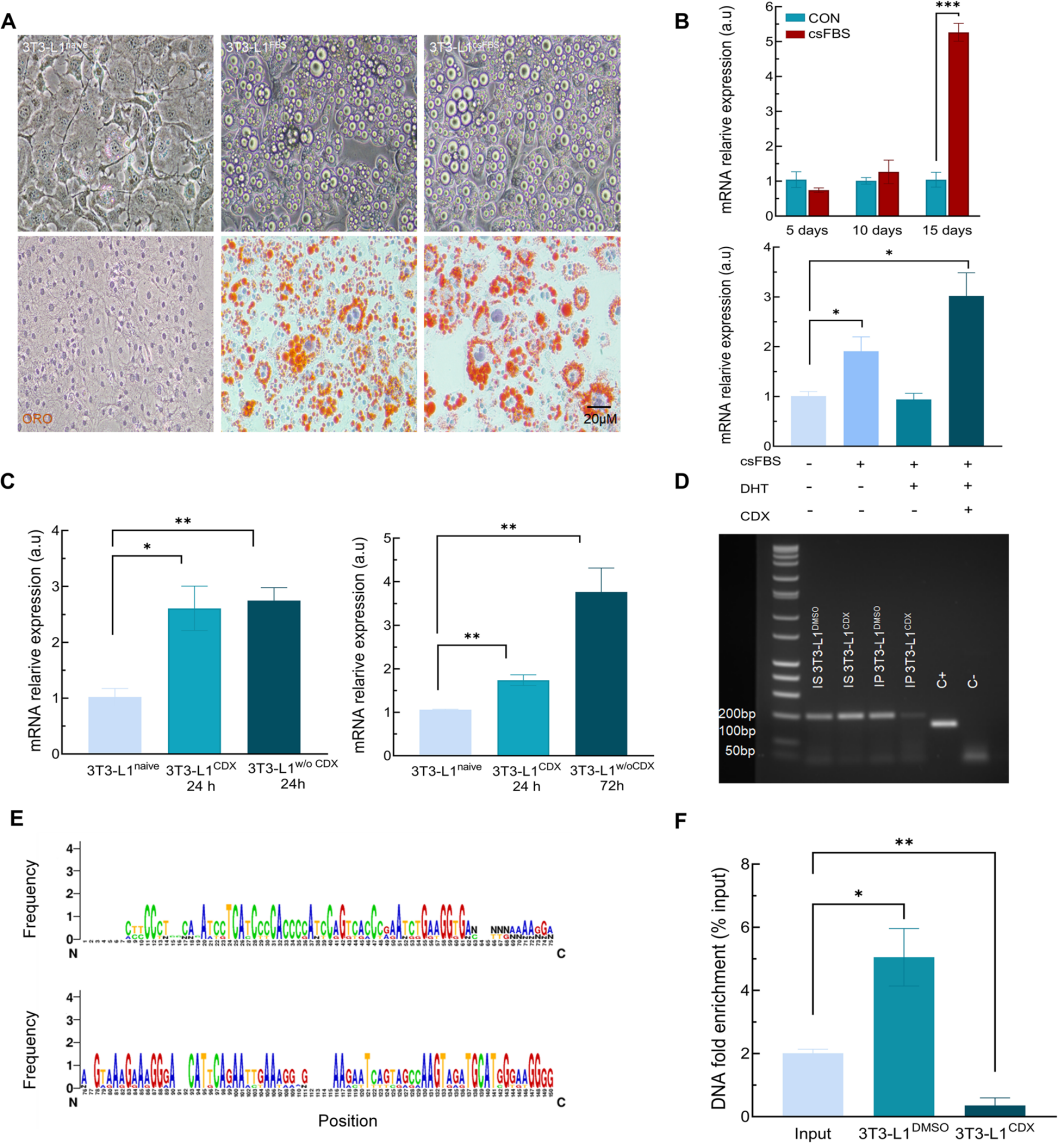
AR acts as a direct regulator of Ucp1 expression in the 3T3-L1 cells in vitro. **A** adipogenic differentiation of 3T3-L1 cultured in undifferentiated condition (naïve), differentiated with normal serum (FBS) and differentiated with steroid-depleted serum (csFBS). Upper images show phase contrast of three experimental groups. Lower images show neutral lipid staining with ORO. Images were taken at 200X of magnification. **B** In Ucp1 expression levels under steroid-depleted FBS for 5,10 and 15 days (upper panel). Ucp1 expression under steroid-depleted FBS, androgen replacement by 5 nM DHT and specific blockade of AR by 20 µM bicalutamide (CDX, lower panel). **C** Ucp1 expression after incubation for 24 h with CDX and withdraw for 24 h (left) and 72 h (right). **D** 2% agarose gel of PCR products derived from ChIP. (IS) input sample, (IP) immunoprecipitated sample, (DMSO) vehicle, (CDX) bicalutamide. **E** Sequence alignment of ChIP results vs complete Ucp1 sequence. Scheme represents which nucleotide correspond to every position. (A) adenine, (C) cytosine, (G) guanin, (T) thymine, (N) non detectable. Frequency represents the conservation of this position between sequences. Symbol height represents the relative frequency for this nucleotide in the predicted position. **F** DNA fold enrichment vs input after immunoprecipitation in 3T3-L1 fibroblast incubated with vehicle (DMSO) or 20 µM bicalutamide (CDX). *n* = 3. Data were represented as mean ± SEM. * *p* < 0.05, ** *p* < 0.01, *** *p* < 0.001

Such 3T3-L1 cells were differentiated into adipocyte-like cells by using a pharmacological cocktail containing IBMX, dexamethasone, RSGZ, and insulin for 10 days. After differentiation, the effect of androgen deprivation for 15 days was assayed. Adipocyte-like differentiation was confirmed by the presence of large, oil red-positive, lipid droplets in the differentiated 3T3-L1 cells, as compared to naïve, control 3T3-L1 (Fig. 6A). No apparent morphological differences between differentiated cells cultured either in the presence or in the absence of androgens were found (Supplementary Fig. 5A). Interestingly, a significant increase in *Ucp1* mRNA expression was found in cells incubated in androgen-deprived csFBS supplemented media. The addition of dihydrotestosterone (DHT) to the culture media totally restored the *Ucp1* expression to control levels. Furthermore, incubation with the antiandrogen bicalutamide (Casodex, CDX) significantly increased *Ucp1* expression, likewise csFBS (Fig. 6B).

To verify whether the increment in *Ucp1* expression was reversible, cells were incubated for 24 h with CDX and then cultured with complete media without CDX for additional 24 or 72 h. As can be observed (Fig. 6C), the removal of CDX for 24 or 72 h did not diminish the levels of *Ucp1* mRNA, suggesting that such increased expression of *Ucp1* after androgen removal is a long-term event.

### AR inhibits Ucp1 expression by a direct interaction with the Ucp1 promoter

Previous results prompted us to study whether AR downregulates directly the expression of *Ucp1* in 3T3-L1 cells by transcriptional activity. To confirm this point, the interaction between the *Ucp1 *gene promoter and AR was determined by ChIP assay in 3T3-L1 fibroblasts incubated with CDX for 24 h. A PCR product of roughly 200 bp was detected in all samples. PCR product found in fibroblasts incubated with the vehicle (IP 3T3-L1^DMSO^) was like that of samples without immunoprecipitation (IS 3T3-L1^DMSO/CDX^). However, samples incubated with CDX and immunoprecipitated for 24 h (IP 3T3-L1^CDX^) have much less PCR product when compared to samples incubated with vehicle (0.1% DMSO) (Fig. 6D). Additionally, PCR product was sequenced, and multiple sequence alignment was performed. Sequences were aligned to the complete mouse *Ucp1* gene (#NM_009463) (Fig. 6E). Finally, the relative enrichment of *the Ucp1* promoter was analyzed by qPCR after CDX incubation. Samples incubated with vehicle presented high levels of enrichment compared to the input sample. On the contrary, the sample incubated with CDX for 24 h showed significantly lower levels of enrichment than the input sample (Fig. 6F).

### Secretome from differentiated 3T3-L1 cells alters prostate cancer cells proliferation

**Fig. 7:**
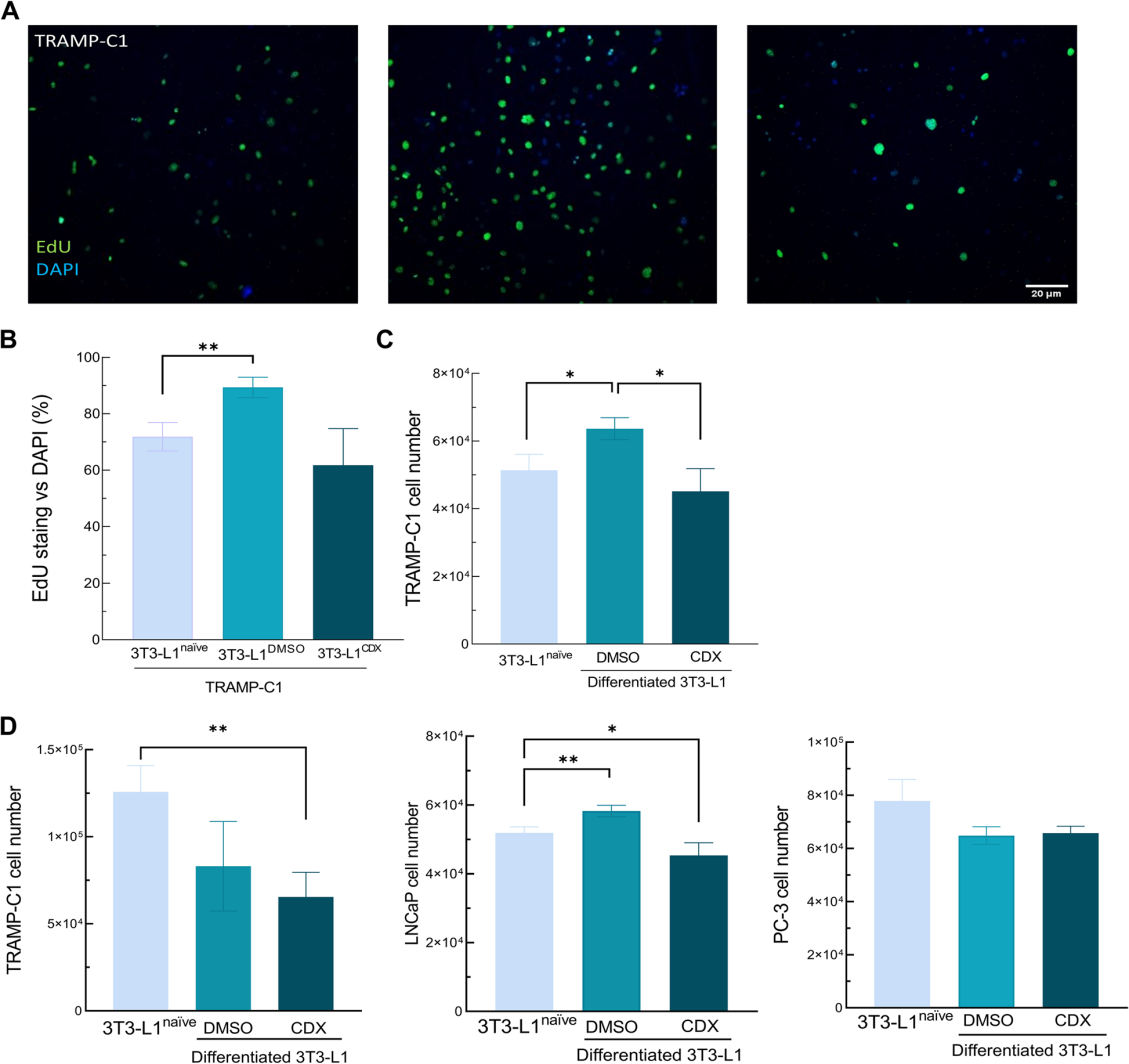
Effect of 3T3-L1 secretome on the proliferation of PCa cells in vitro. **A** Edu labeling of proliferating TRAMP-C1 cells after co-culture assay in Transwell.™ inserts for 48 h with 3T3-L1 undifferentiated cells (naïve), differentiated in presence of androgen signaling (DMSO) or without androgen signaling (CDX). Images were taken at 100X of magnification. **B** Quantification of EdU positive cells vs DAPI staining cells in the same groups as previously described. **C** TRAMP-C1 cell number after incubation 48 has the same condition in the previous graph. **D** Cell number quantification of TRAMP-C1, LNCaP and PC3 cells incubated with 3T3-L1 derived EVs isolated from undifferentiated fibroblast or differentiated in presence of DMSO or CDX. *n* = 3. Data were represented as mean ± SEM. * *p* < 0.05, ** *p* < 0.01

To confirm a role for brown/beige adipocytes on tumor growth, our next approach was to analyze the role of the secretome from differentiated 3T3-L1 cells in PCa cell proliferation. With this aim, co-culture assay was performed using transwell™ inserts and proliferative TRAMP-C1 cells were quantified by EdU labeling. Number of EdU-positive cells was significantly higher in the group co-cultured with 3T3-L1 adipocyte-like cells, compared to TRAMP-C1 cells incubated with naïve 3T3-L1 fibroblast or 3T3-L1 adipocyte-like cells incubated with CDX (Fig. 7A, B).

Proliferation was also analyzed by carrying out experiments using conditioned media from differentiated cells in the presence or absence of antiandrogen CDX for 48 h. After 48 h the same results were observed, but in this case the presence of CDX in the differentiated media of 3T3-L1 adipocyte-like cells prevented the increase in proliferation of TRAMP-C1 cells cultured in media derived from cells incubated with standard differentiated 3T3-L1 containing androgens (Fig. 7C).

As an endocrine tissue, adipocytes release a high quantity of extracellular vesicles (EVs) which might contain key regulatory bioactive compounds targeting other cells. EVs were isolated from either naïve 3T3-L1 fibroblasts or differentiated 3T3-L1 adipocyte-like cells in the presence or absence of CDX and *Ucp1* expression was used as positive control of brown/beige differentiation. As previously shown, CDX incubation significantly increased *Ucp1* expression in differentiated 3T3-L1 cells after 7 days (Supplementary Fig. 6A). Once differentiation was confirmed, EVs were isolated by differential ultracentrifugation, and size and aggregation were measured by DLS, showing a ranging size between 30 and 600 nm, which corresponds to the described values for EVs [19]. Interestingly, EVs derived from 3T3-L1 adipocyte-like cells incubated with CDX have a larger size (196.69 ± 37.04) than EVs obtained from 3T3-L1 fibroblasts (131.79 ± 24.24) or 3T3-L1 adipocyte-like cells (125.49 ± 25.09) (Supplementary Fig. 6B).

To confirm whether EVs derived from differentiated 3T3-L1 cells with or without CDX have modulatory activity in the proliferating cancer cells, murine and human androgen-dependent PCa cells (human LNCaP and murine TRAMP-C1) and human androgen-independent cells (PC-3) were incubated for 48 h with EVs derived from fibroblasts or 3T3-L1 adipocyte-like cells. For the assay, 5 µg of protein from the EVs of each sample was employed.

An increase of LNCaP cells proliferation was found after 48 h of incubation with 3T3-L1 adipocyte-like derived EVs. On the contrary, the proliferation of cells incubated with EVs from 3T3-L1 adipocyte-like cells previously incubated with CDX was significantly reduced. In the case of TRAMP-C1 cells, incubation with EVs from differentiated 3T3-L1 cells in presence of CDX significantly reduced cell number compared to TRAMP-C1 cells incubated with EVs from naïve fibroblasts. EVs derived from adipocyte-like cells did not alter PC3 proliferation (Fig. 7D).

### Adipose tissue influences the growth of prostate tumor xenografts

Since neither castration nor RSGZ treatment discriminated the direct effect of brown/beige adipose tissue on the prostate tumor progression in vivo, a xenograft experiment was carried out. First, WAT from a donor mouse was cultured in vitro in either undifferentiating conditions or in others that induced further differentiation into a brown/beige phenotype. After three weeks of culture, differentiated adipose tissue fragments displayed a characteristic brown/beige color, while undifferentiated WAT maintains its regular aspect (Supplementary Fig. 6A). Furthermore, expression levels of *Ucp1* revealed that the WAT fragment incubated with a cocktail containing dexamethasone, IBMX, indomethacin, RSGZ, and T3 hormone had a significant increase of *Ucp1* expression, thus confirming browning (Supplementary Fig. 6B).

Once the differentiation was determined, adipose tissue fragments were implanted in the right and left flanks of host mice. One week after adipose tissue implantation, 2 × 10^6^ TRAMP-C1 cells were inoculated by direct injection in the adipose tissue and 1-month later mice were sacrificed, and blood and tumors were collected.

**Fig. 8:**
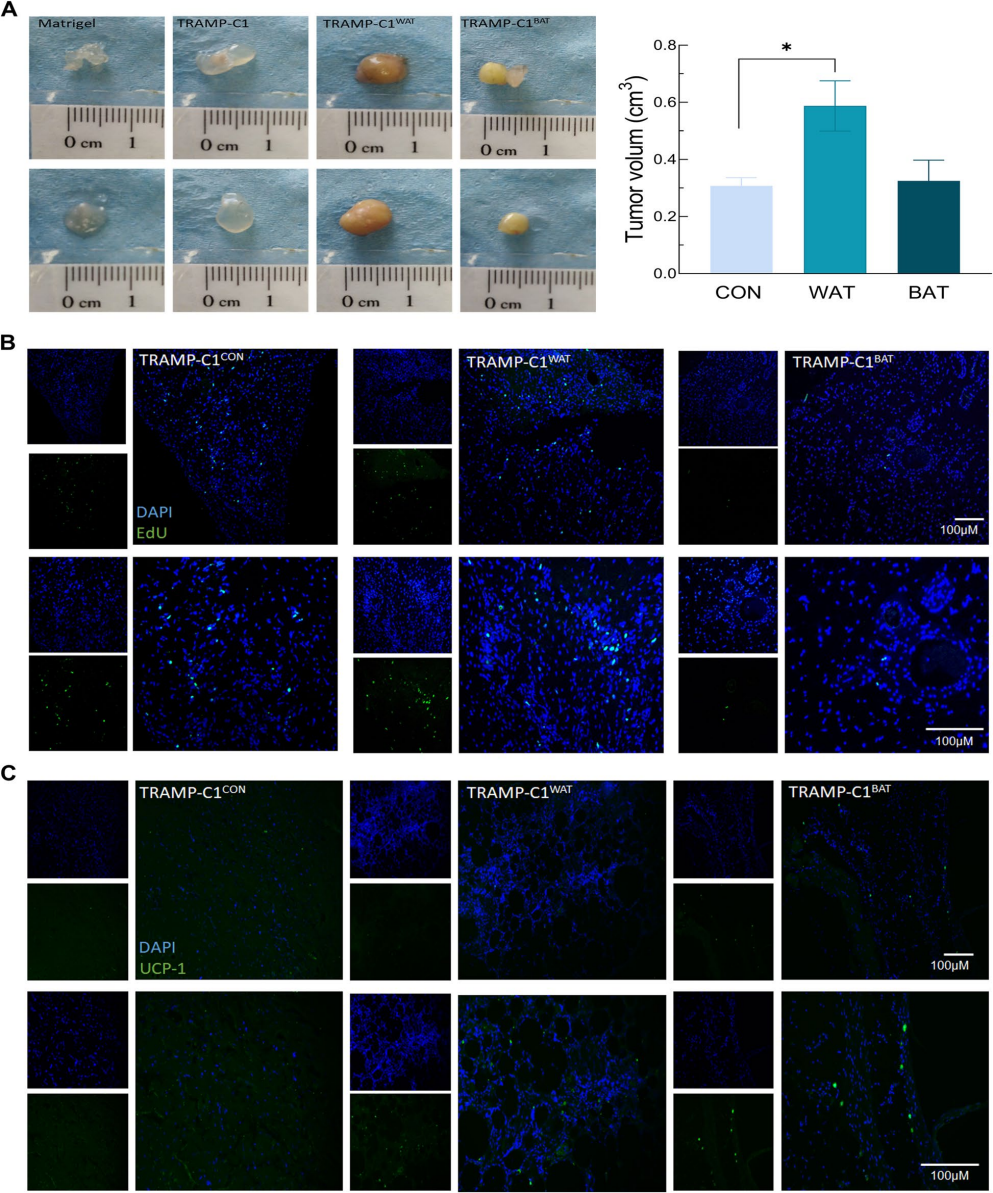
Allograft of in vitro transdifferentiation of WAT do not increase TRAMP-C1 proliferation in vivo. **A** Ex vivo images of TRAMP-C1 tumors allograft 4 weeks after the inoculation (left). Variation of in vivo tumor volume at the end point between, naïve TRAMP-C1 cells (CON), TRAMP-C1 inoculated with WAT (WAT) or TRAMP-C1 inoculated with transdifferentiated fat (BAT). **B** EdU staining in the allografts. Images were taken at 100 and 200X of magnification. **C** UCP1 protein production in the allografts in the three different groups Images were taken at 100 and 200X of magnification. Tumors per group *n* = 4.Data were represented as mean ± SEM. * *p* < 0.05

Three weeks after the implantation, tumor volumes of TRAMP-C1 cells injected in WAT fragments (TRAMP-C1^WAT^) were significantly higher than tumors derived from TRAMP-C1 cells injected in Matrigel (TRAMP-C1^naïve^) while the growth of TRAMP-C1 cells injected in brown/beige fragments (TRAMP-C1^BAT^) was equal to controls and showed an almost significant reduction compared to that of cells injected in WAT fragments. Furthermore, ex vivo images showed that TRAMP-C1^WAT^ tumors had harder consistency and slightly higher volume than TRAMP-C1^naïve^ and TRAMP-C1^BAT^ tumors (Fig. 8A). This was corroborated by the observation of cell proliferation by EdU labeling, showing a higher proliferation in TRAMP-C1^WAT^ tumors than in TRAMP-C1^Naïve^, while in TRAMP-C1^BAT^ tumors displayed a scarce number of proliferative cells, even lower than controls (Fig. 8B). As predicted, TRAMP-C1^BAT^ adipose tissue produced UCP1, while in TRAMP-C1^WAT^ explants no UCP1 was detected (Fig. 8C). Interestingly, TRAMP-C1^WAT^-derived tumors, tumor cells infiltrated the adipose tissue fragment, while in TRAMP-C1^BAT^ tumors no infiltrating cells were observed (Supplementary Fig. 6E).

## Discussion

While some PCa tumours progress slowly and positively respond to antiandrogenic treatments during the first stages, several patients experience undesired tumour relapses, thus acquiring a hormone-resistant phenotype, a further stage associated with hight morbidity and mortality. Despite numerous advances and a great deal of scientific efforts trying to elucidate the underlying mechanisms of this transition to androgen independence, there still needs to be more evidence in this regard. Nonetheless, the importance of tumour microenvironment throughout the progression has been widely recognized [20].

As a major component of the tumor microenvironment, adipocytes/adipose tissue exert crosstalk between PPAT and prostate cells, thus influencing the phenotype and behavior of tumor cells [21]. The positive relationship between obesity and cancer, including PCa, has motivated numerous studies [22], most focused on the intercellular communication between white adipocytes and tumor cells [23]. The role of WAT has been the common target of study, and brown adipose tissue has received much less attention. BAT has recently emerged as a novel strategy for reducing adiposity and regulating homeostasis, and in this context, some beneficial effects have been described in metabolic pathologies such as diabetes [24], but a general role of BAT in tumour progression, particularly in PCa has not been previously addressed. To our knowledge, this is the first report showing that castration increases the amount of BAT within PPAT in either WT or TRAMP mice.

The effect of androgens removal on fat content has been widely reported [25–27]. Castration of mice results in a reduced body weight and an increase in adipose tissue, concomitant with an increase in body temperature [28–30], which differs from what occurs in humans [31] or the classically reported increase in body weight observed in domestic pets such as cats and dogs [32–34]. In the case of livestock, castration is an ancient strategy to increase meat production and quality [35]. In all cases, castration usually accounts for a reduced metabolic rate and increased body temperature, though. An increase in WAT depots is inversely related to metabolic rate, which, in turn, is associated with the lack of circulating testosterone, reducing energy demand [36]. However, in the study presented here, we did not observe a decrease in energy expenditure after castration in WT animals, probably because animals were sacrificed shortly after orchiectomy (4–8 weeks).

Interestingly, tumour bearing TRAMP mice undergoing orchiectomy display a higher metabolic and energetic demand. In this pathologic situation, adipose tissue could serve as an energy source that contributes to fueling tumor growth, as it has been proposed by other groups [37]. This is supported by the positive correlation between intratumor adipocytes and tumor progression [29–31]. In this same cancer model, Huang et al. (2018) have demonstrated that invasive tumour borders affect adipose tissue, leaving small groups of adipocytes included within the tumor mass [38]. Using other murine models, it has been demonstrated that adipose stromal and endothelial cells from systemic circulation are recruited by cancer cells, and in turn promote tumour growth [39]. However, castration changed PPAT-WAT by inducing the occurrence of UCP1-positive, multilocular, beige-like adipocytes. Furthermore, WAT adipocytes in the vicinity of multilocular depots also displayed UCP1 immunostaining, suggesting a possible trans-differentiation process from WAT into BAT. A few, primarily descriptive, examples reporting this androgen removal-derived trans-differentiation has been reported, but no insights into the molecular mechanisms involved in the process have been confirmed [28].

In our model, exogenous testosterone restores to normal the brown/beige fat depots in the PPAT of TRAMP-castrated mice, as well as the expression of *Ucp1*, thus confirming the androgenic control of the mechanism*.* An androgen control has also been observed in hypogonadal male mice, in which testosterone reduced WAT depots and increased BAT content [40]. On the contrary, Gasparini et al. (2019) have recently reported that castration increases iBAT, while testosterone reverses the effect through a mechanism of adipocyte sensitization to glucocorticoids [41]. Even though conflictive, these results highlight the importance of the endocrine control of WAT/BAT by androgens, thought the specific effect has not been yet determined. Nevertheless, data here suggest that tissue specificity might be relevant in the effect of androgens on WAT/BAT balance.

The thermogenic activity of castration-induced brown/beige fat was confirmed by the observed increase in body temperature, which was prevented by testosterone injection. Castration-induced browning is not restricted to the PPAT but instead seems to play a systemic role in WAT browning, in agreement with results recently published [28], as supported by the changes in body temperature observed, indicating the metabolic shift. However, while the metabolic rewiring induced by testosterone depletion it is well known, the extent of the specific role of BAT in this metabolic shift has yet to be determined.

Chronic inflammation is a major risk factor underlying tumorigenesis in PCa [42, 43]. It is well known that excessive adipose tissue growth has a direct role in regulating immune populations within the PCa microenvironment [44]. In the study shown here, castration increased the mast cell population within the prostate tumor microenvironment. Only a few studies have addressed the role of androgens on mast cells, but previous results demonstrated an active role of testosterone an activator of mast cell degranulation [45, 46]. More recently, MacKey and co-workers have shown a protective role of testosterone in mast cell-associated disease by reprograming bone marrow precursors [47]. Macrophages are frequently found close to adipose tissue, in the proximity of tumours [48], though they have been more extensively studied than mast cells. Under physiological conditions, macrophages represent approximately 5% of total immune cells. Nevertheless, macrophage infiltration increases significantly within tumor tissues, up to roughly 50% of whole immune cells in the microenvironment [48, 49]. There is a direct correlation between the increase in the number of macrophages and PCa progression and poor outcome, as well as the acquisition of a castration-resistant phenotype [50]. Our results confirm that TRAMP mice display more macrophages than WT mice surrounding the secretory tissue, and castration reduces their number, in agreement with other studies carried out in both patients and animal models. Cancer and metabolic diseases promote WAT-derived inflammation, displaying a higher infiltration of the proinflammatory M1 macrophage population. On the contrary, WAT browning tends to reduce adipose tissue-associated inflammation, increasing the ratio of M2-polarized macrophages with an anti-inflammatory profile [51]. This was confirmed here, where the high dependence of androgens by the prostate lead to a reversed situation after castration regarding the number of macrophages. However, no differences in M1/M2 populations were found in castrated vs. intact, transgenic TRAMP mice.

A reduction in WAT has also shown beneficial effects on other pathologies [22, 23, 43]. In the prostate, however, it is difficult to determine the exact impact of brown/beige adipose tissue on PCa progression due to the specific antiproliferative effect of androgen withdrawal in this tumour. The attempt of pharmacological induction of browning using RSGZ treatment for ten days, which is sufficient to induce the appearance of brown/beige adipose tissue in subcutaneous and visceral fat [17], did not result in a significant difference in brown/beige fat levels in the prostate tumor microenvironment of TRAMP mice. Even more, we found an undesired side effect of RSGZ on tumor growth, which did not allow us to decipher the actual role of browning on the growth of tumor glands. Previous findings suggest a pivotal role of PPARγ in tumor progression. Thus, while its overexpression inhibits colon cancer cell proliferation [52], PPARγ agonists suppress melanoma growth in mice [53]. However, opposite results have been obtained in this same tumor [54]. Results here would confirm recent evidence suggesting that PPARγ is generally associated with a poor prognosis in prostate cancer [55], since it is related to an increase in AKT expression and further mitogenesis [56] or other AR-dependent and independent mechanisms [57]. All together, these conflictive results indicate a complex role of this nuclear receptor in cancer biology that needs further clarification and might be dependent on the type of cancer and tumor stage.

AR is broadly expressed in both WAT and BAT. Interestingly, the subcellular location of AR changes between the two types of fat cells, which agrees with results previously published in vitro [58]. This location shift, along with the increase in *Ucp1* expression following androgen deprivation, supports a direct role of AR on the *Ucp1* promoter, as it was confirmed by Chipo assay. AR is a repressor of several genes related to adipogenic function, including *PPARɣ* or *C/EBPα* [59, 60]. Our data demonstrated that the *Ucp1* gene is directly repressed by AR, suggesting its participation at different stages during the browning. Therefore, WAT transdifferentiation into brown/beige adipose tissue could be partially controlled at the endocrine level in PPAT, with the corresponding clinical implications after antiandrogenic treatments.

In this context, the endocrine effect of the adipose tissue within the tumor microenvironment through the secretome is well documented [61]. To test the impact of WAT/BAT secretome, we incubated mouse epithelial TRAMP-C1 cancer cells with conditioned media from 3T3-differentiated adipocyte-like cells, showing promotion of cell proliferation on androgen-independent TRAMP-C1 cells. Similar effects have been as previously reported using either TRAMP-C1 spheroids and estrogen-dependent MCF-7 and estrogen-independent MDA-MB-231 breast cancer cells [62]. Small vesicles have gained attention as an intercellular communication mechanism, and their role on tumor microenvironment is widely investigated. Most adipokines released by adipocytes are contained in EVs, and they exert critical roles related to most of the hallmarks of cancer, favoring the acquisition of the tumor phenotype [9]. EVs derived from adipocytes previously deprived of androgens significantly reduced PCa proliferation, while the incubation with EVs from white adipocytes increases the proliferation rate. In addition to the direct antitumor effects of BAT, WAT browning might be responsible for reducing adiposity and the subsequent reduction of the protumor activity of WAT-derived EVs.

The direct role of browning on PCa cells was finally tested using WAT and BAT organotypic cultures to engraft them with TRAMP-C1 cancer cells. Results obtained agree with those published by Blumenfield et al. (2018), who found that tumors inoculated with WAT explants grew significantly faster than TRAMP-C1 naïve tumors [18]. Even more, tumours inoculated with androgen-depleted WAT and showing markers of BAT exhibit no difference with controls.

## Conclusions

In this work, it is concluded that androgens removal promotes the appearance of BAT in the PPAT. Moreover, AR has a key role differentiating WAT to BAT and AR regulates *Ucp1* expression in vitro. Also, it is concluded that the secretome of BAT adipocytes reduces the proliferation of PCa cells in vitro and the tumor allograft in vivo*,* suggesting that the trans-differentiation of WAT to BAT could be a new strategy to prevent the proliferation of the prostate cancerous cells promoted by the presence of WAT in the tumor microenvironment*.*

Findings reported here support the idea that androgen withdrawal promotes WAT browning and this brown/beige adipose tissue exerts a completely different set of effects on prostate cancer cells, contrary to WAT. This may indeed have some clinical implications for tumour treatment, not only in PCa but also other tumours. In the case of the prostate, BAT/beige -likely coming from transdifferentiated WAT after androgen withdrawal- would be responsible of: i) Reducing the positive enhancement of tumours by WAT; ii) Reducing cancer cells growth directly by mechanisms of cell communication; iii) Reducing tumour progression by changing cell populations or communication within the tumour microenvironment. Whether brown adipocytes per se might have their role in modulating tumour growth in the presence or absence of androgens requires further research but here we proposed that they could be beneficial even in hormone-resistant tumours. Insights into the impact of BAT on cancer growth deserves attention. However, pharmacological drugs which triggers browning, including RSGZ used in this study, frequently display undesired side effects. Therefore, it is noteworthy seeking for new tools to induce browning.

### Supplementary Information

**Additional file 1:** **Table S1.**

**Additional file 2:** **Table S2.**

**Additional file 3: Table S3.**

**Additional file 4:Figure S1. **(A) Histology of the prostate of WT and TRAMP mice after 4 and 12 weeks of castration. Images were taken at 400X of magnification (left). GU vs body weight ratio of WT and TRAMP mice after 4 and 12 weeks of castration *n*=10 (right). (B) Prostate epithelium proliferation by labeling with EdU (left) and quantification of EdU positive cells vs DAPI surface (right) in WT and TRAMP mice after 4 weeks of castration. Five images from each mouse were used for the quantification. (C) Effect of castration (CAST) and 2.5mg/Kg testosterone administration (CAST+TEST) on the prostate epithelium of TRAMP mice (left), images were taken at 100 and 400X of magnification. GU vs body weight ratio in the same groups (right) *n*=5. Data were represented as mean± SEM. * *p*<0.05,** *p*<0.01, *** *p*<0.001.

**Additional file 5:Figure S2. White adipocytes were secluded to the tumor mass during tumor progression**. Images of intratumoral adipocytes in the prostate of TRAMP mice during tumor progression (upper). White adipocyte cell number and average area in the three different pathologies of the prostate, well differentiated (WD), moderately differentiated (MD) and poorly differentiated (PD). Images were taken at 100 and 400X of magnification. For the quantification of adipocyte number 3 different slides were analyzed. For the quantification of adipocyte average area 100 cells were quantified (lower). Muse number per group: *n*=10- Data were represented as mean ± SEM. Non detectable (ND). *** *p*<0.001.

**Additional file 6:Figure S3.** AR expressionin prostate epithelial cells in TRAMP and TRAMP castrated (CAST) for 12 weeks. Images show the expression of AR (red) in the prostate of TRAMP and TRAMP castrated mice. Images were taken at 100x and 200x of magnification.

**Additional file 7:Figure S4.** Strategies that produce brown/beige fat in the PPAT did not alter macrophage polarization. (A) Macrophage polarization in the prostate microenvironment of WT and TRAMP mice after castration for 12 weeks. CD86 and CD11c (M1 macrophage markers), CD206, arginase1 (M2 macrophage markers). Images were taken at 400X of magnification.

**Additional file 8:Figure S5.** Effect of the androgen signaling blockade on the differentiation of 3T3-L1 cell***. ***Morphometric analysis of 3T3-L1 lipid droplets under undifferentiated (naïve), differentiated with FBS (FBS) and with steroid-depleted FBS (csFBS). Quantification of lipid droplet number (left), lipid droplet average area (center) and ORO absorbance (right 100 lipid droplets were used for the all the quantifications). *n*=3. Data were represented as mean ± SEM. Non detectable (ND), ** *p*<0.01, ***p<0.001, **** *p*<0.0001.

**Additional file 9: Figure S6.** Effect of the androgen signaling blockade on the EVs production in 3T3-L1 cells***. ***(A) Analysis of Ucp1 expression of 3T3-L1 cells used for EVs isolation *n*=3. (B) Size measurement by DLS of the 3T3-L1 derived EVs using the previous groups *n*=3. Data were represented as mean ± SEM. **** *p*<0.0001.

**Additional file 10:Figure S7.** Study of adipose tissue after the differentiation procedure in vitro and the allograft. (A) Images of adipose tissue explants after 3 weeks of in vitro culture. control (CON) and transdifferentiated (COCKTAIL). (B) Ucp1 expression of adipose tissue explants after the transdifferentiation procedure. (C) Micrographs of TRAMP-C1 tumor cells allograft. Arrows show TRAMP-C1 cell infiltration. Images were taken at 40, 100 and 200X of magnification. Data were represented as mean ± SEM. * *p*<0.05,** *p*<0.01.

## Data Availability

The datasets used and/or analyzed during the current study are available from the corresponding author on reasonable request.

## Abbreviations

AR: Androgen receptor
AUC: Area under the curve
BAT: Brown adipose tissue
BCA: Bicinchoninic acid
Bw: Body weight
C/EBPα: CCAAT-enhancer-binding proteins alpha
CAST: Castrated
CDX: Casodex/bicalutamide
ChIP: Chromatin immunoprecipitation
CO_2_: Carbon dioxide
csFBS: Charcoal-stripped fetal bovine serum
DAPI: 4’,6-Diamidino-2-phenylindole
DHEA: Dehydroepiandrosterone
DHT: Dihydrotestosterone
DLS: Dynamic light scattering
DM: Differentiation medium
DMSO: Dimethylsulfoxide
EdU: 5-Ethynyl-2-deoxyuridine
EE: Energy expenditure
EVs: Extracellular vesicles
eWAT: Epididymal white adipose tissue
FBS: Fetal bovine serum
FITC: Fluorescein isothiocyanate
GU: Genitourinary
iBAT: Interscapular brown adipose tissue
IBMX: 3-Isobutyl-1-methylxantine
IP: Immunoprecipitated
IS: Input sample
MD: Moderately differentiated
O_2_: Oxygen
ORO: Oil red O
PB: Phosphate buffer
PBS: Phosphate buffered saline
PCa: Prostate cancer
PD: Poorly differentiated
PFA: Paraformaldehyde
PM: Proliferative medium
PPAR-ɣ: Peroxisome proliferator-activated receptor gamma
PPAT: Periprostatic adipose tissue
RER: Respiratory exchange ratio
RSGZ: Rosiglitazone
RT: Room temperature
scWAT: Subcutaneous white adipose tissue
SEM: Standard error of the mean
TEST: Testosterone
TRAMP: Transgenic adenocarcinoma of the mouse prostate
UCP1: Uncoupling protein 1
VCO_2_: Carbon dioxide volume
VO_2_: Oxygen volume
WAT: White adipose tissue
WD: Well differentiated
WT: Wild type
