# Supplementary material for: Castration promotes the browning of the prostate tumor microenvironment
Source: Cell Commun Signal. 2023 Sep 28;21:267. doi: 10.1186/s12964-023-01294-y (PMC10536697; doi:10.1186/s12964-023-01294-y)
Supplement: Supplementary file 2 — Additional file 1: Table S1. [file 12964_2023_1294_MOESM1_ESM.docx]

**Supplementary table 1. List of chemical reagents and suppliers used in this study.**

| REAGENT | IDENTIFIER | SOURCE |
| --- | --- | --- |
| Chemical reagents | | |
|  | | |
| 3-isobutyl-1-methylxanthine | 410957 | Sigma-Aldrich, Merck |
| 4-6-Diamino-2-Phenylindole | D1306 | Invitrogen, Thermo Fisher Sci. |
| Agarose E | 8100 | Condalab |
| Bovine Serum Albumin | A1391 | Panreac Química SLU |
| Antibiotic and antimycotic cocktail | 15240062 | Gibco^TM^, Thermo Fisher Sci. |
| Sodium ascorbate | A7631 | Sigma-Aldrich, Merck |
| Bicalutamide | B9061 | Sigma-Aldrich, Merck |
| Charcoal | 1021860250 | Sigma-Aldrich, Merck |
| Chlorofom | 25668 | Sigma-Aldrich, Merck |
| Dexamethasone | D9184 | Sigma-Aldrich, Merck |
| Dextran-T70 | D1390 | Sigma-Aldrich, Merck |
| D-glucose | 0188 | AMRESCO Inc. |
| Dehydroepiandrosterone | 390089 | Sigma-Aldrich, Merck |
| Dihydrotestosterone | A8505 | Sigma-Aldrich, Merck |
| Dimethyl sulfoxide | 1,319,541,611 | AppliChem GmbH |
| DMEM | D5671 | Sigma-Aldrich, Merck |
| DMEM-F12 | D8437 | Sigma-Aldrich, Merck |
| EDTA | 245 | AMRESCO Inc. |
| Ethanol | ALCH0121F5 | VWR |
| EUKITT® | 6,272,068 | ORSAtec GmbH |
| FBS | 11573397 | Gibco^TM^, Thermo Fisher Sci. |
| Fluoromount^TM^ | F4680 | Sigma-Aldrich, Merck |
| Nuclease free H_2_O | AM9938 | Invitrogen, Thermo Fisher Sci. |
| Hematoxilin | 1092530500 | Sigma-Aldrich, Merck |
| HEPES | H3375 | Sigma-Aldrich, Merck |
| Indomethacin | ACRO458030250 | VWR |
| Human insulin | I9272 | Sigma-Aldrich, Merck |
| 2-propanol | I9516 | Panreac Química SLU |
| Ketamine | N/A | Merial |
| L-Glutamine | 67513 | Sigma-Aldrich, Merck |
| NaOH | 1.06498 | Sigma-Aldrich, Merck |
| NBCS | 1158050 | Gibco^TM^, Thermo Fisher Sci. |
| Oil Red O | 21600 | Sigma-Aldrich, Merck |
| Paraformaldehyde | A3813,0500 | Applichem GmbH |
| Sodium piruvate | 11360-039 | Gibco^TM^, Thermo Fisher Sci. |
| Pronase | 10165921001 | Sigma-Aldrich, Merck |
| Rosiglitazone | CAYM71740 | Cayman Chemicals |
| RPMI | R0883 | Sigma-Aldrich, Merck |
| sodium citrate | 71365 | Sigma-Aldrich, Merck |
| Tripsin-EDTA 0.05% | 25300054 | Gibco^TM^, Thermo Fisher Sci. |
| Goat serum | 16210064 | Gibco^TM^, Thermo Fisher Sci. |
| Nu-serum IV complement | 392-0321 | Corning® |
| Testosterone | T1500 | Sigma-Aldrich, Merck |
| TRI Reagent® | T9424 | Sigma-Aldrich, Merck |
| triiodothyronine | 11946891 | Thermo Fisher Sci. |
| Tritón X-100 | 327371000 | VWR |
| Trizma base | 28808.294 | VWR |
| Tween 20 | P7949 | Sigma-Aldrich, Merck |
| Xilacine | N/A | Bayer |
| Comercial kits | | |
| BCA protein detection kit | KB03005 | Bioquochem |
| *In vivo* EdU Click Kit 488 | BCK488-IV-IM-S | Baseclick GmbH |
| MyTaq^TM^ HS Red Mix | BIO25047 | Bioline, Meridian Bioscience |
| NG dART RT-PCR kit | E0802-02 | EURx Sp. z.o.o |
| Power Up^TM^ SYBER^TM^ Green Master Mix | A25742 | Applied Biosystem |
| SimpleChIP® Enzymatic Chromatin IP | 9003 | Cell signaling Technology |
| Equipment | | |
| Transwell® inserts | 734-1567 | Corning® |
| Biotek Synergy H1 | N/A | BioTek Instruments |
| Nikon DS-5M camera | N/A | Nikon |
| Nanodrop® 2000 | N/A | Thermo Fisher Sci. |
| Ultraturrax homogenizer | N/A | IKA-Werke |
| Nikon Eclipse 80i | N/A | Nikon |
| Swing Bucket SW 40 Ti | N/A | Beckman Coulter |
| MyCycler^TM^ Thermalcycler | N/A | Bio-Rad |
| QuantiStudio 5 Thermalcycler | N/A | Applied Biosystem |
| Beckman Optima L90K ultracentrifuge | N/A | Beckman Coulter |
| Nikon DS-L1 control unit | N/A | Nikon |
| Zetasizer Nano | N/A | Malvern Panalytical |
| Software | | |
| FinchTV | N/A | https://digitalworldbiology.com/FinchTV |
| ImageJ/Fijji | N/A | https://imagej.nih.gov/ij/ |
| Mega11 | N/A | https://www.megasoftware.net/ |
| PerlPrimer | N/A | http://perlprimer.sourceforge.net/ |
| Prism8 | N/A | GraphPad Software |
| ZEN 2.6 Pro | N/A | ZEISS |
