# Supplementary material for: Castration promotes the browning of the prostate tumor microenvironment
Source: Cell Commun Signal. 2023 Sep 28;21:267. doi: 10.1186/s12964-023-01294-y (PMC10536697; doi:10.1186/s12964-023-01294-y)
Supplement: Supplementary file 3 — Additional file 2: Table S2. [file 12964_2023_1294_MOESM2_ESM.docx]

| Gene | Forward sequence | Reverse sequence |
| --- | --- | --- |
| *Tg1* | 5’CCGGTCGACCGGAAGCTTCCACAAGTGCATTTA3’ | 5’AGGCATTCCACCACTGCTCCCATTCATC3’ |
| *Tg2* | 5’GCGCTGCTGACTTTCTAAACATAAG3’ | 5’GAGCTCACGTTAAGTTTTGATGTGT 3’ |
| *Ucp1* | 5’AGGCTTCCAGTACCATTAGGT3’ | 5’CTGAGTGAGGCAAAGCTGATTT3’ |
| *β-actin* | 5’GGCTGTATACCCCTCCAT3’ | 5’CCAGTTGGTAACAATGCCATG3’ |
| *Ucp1 promoter* | 5’CCACACGATGCACTCACTTT3’ | 5’CCCCTTCCCATGCATCTACT3’ |

**Supplementary table 2. Primers sequences of genes analyzed in this work.**
