# Supplementary material for: Castration promotes the browning of the prostate tumor microenvironment
Source: Cell Commun Signal. 2023 Sep 28;21:267. doi: 10.1186/s12964-023-01294-y (PMC10536697; doi:10.1186/s12964-023-01294-y)
Supplement: Supplementary file 4 — Additional file 3: Table S3. [file 12964_2023_1294_MOESM3_ESM.docx]

| ANTIBODY | COMPANY | CODE# | AG RETRIEVAL | 1^O^ AB DILUTION | 2^O^ AB DILUTION |
| --- | --- | --- | --- | --- | --- |
| Androgen Receptor (D6F11) XP® Rabbit mAb | Cell signaling | 5153S | Citrate buffer 10 mM pH 6.0, 95 ^o^C, 20 min | 1:100 | 1:200 |
| Androgen Receptor (441) X | Santa Cruz | SC7305X | N/A | N/A | N/A |
| Anti UCP1 antibody produced in rabbit | Merck | U6382 | Pronase 0.05 % in Tris-HCl pH 7.5, 37 ^o^C, 30 min | 1:6000 | 1:250 |
| F4/80 Monoclonal antibody (CI:A3-1) | Invitrogen | MA1-91124 | N/A | 1:1000 | 1:250 |
| Recombinant Anti- Ki67 antibody (SP6) | Abcam | Ab166677 | Citrate buffer 10 mM pH 6.0, 95 oC, 20 min | 1:200 | 1:200 |
| Anti-CD11c D1V9Y rabbit mAb | Cell signaling | #97585 | Citrate buffer 10 mM pH 6.0, 95 ^o^C 20 min | 1:500 | 1:200 |
| Anti-CD-86 E5W6H rabbit mAb | Cell signaling | #19589 | Citrate buffer 10 mM pH 6.0, 95 ^o^C, 20 min | 1:200 | 1:200 |
| Anti-CD206 E55J rabbit mAb | Cell signaling | #24595 | Citrate buffer 10 mM pH 6.0, 95 ^o^C, 20 min | 1:500 | 1:200 |
| Anti-Arginase 1 D4E3M rabbit mAb | Cell signaling | #93668 | Citrate buffer 10 mM pH 6.0, 95 ^o^C, 20 min | 1:200 | 1:200 |
| Alexa fluor 488 goat anti rat | Invitrogen | A11006 | N/A | N/A | N/A |
| Alexa fluor 488 goat anti rabbit | Jackson Immuno Research | 111-545-003 | N/A | N/A | N/A |

***Supplementary table 3. Antibodies used for immunohistochemical assays.***
