# Supplementary figures and images for: Castration promotes the browning of the prostate tumor microenvironment
Source: Cell Commun Signal. 2023 Sep 28;21:267. doi: 10.1186/s12964-023-01294-y (PMC10536697; doi:10.1186/s12964-023-01294-y)

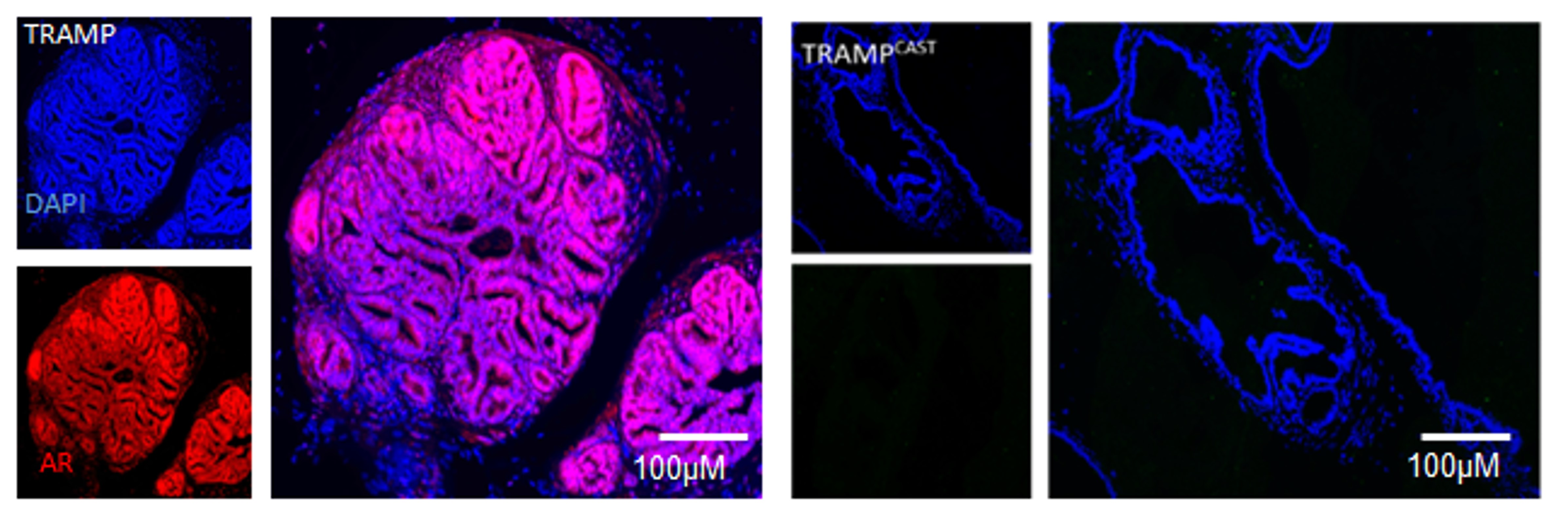

Supplement: Supplementary file 7 — Additional file 6:Figure S3. AR expressionin prostate epithelial cells in TRAMP and TRAMP castrated (CAST) for 12 weeks. Images show the expression of AR (red) in the prostate of TRAMP and TRAMP castrated mice. Images were taken at 100x and 200x of magnification. [file 12964_2023_1294_MOESM6_ESM.jpg]

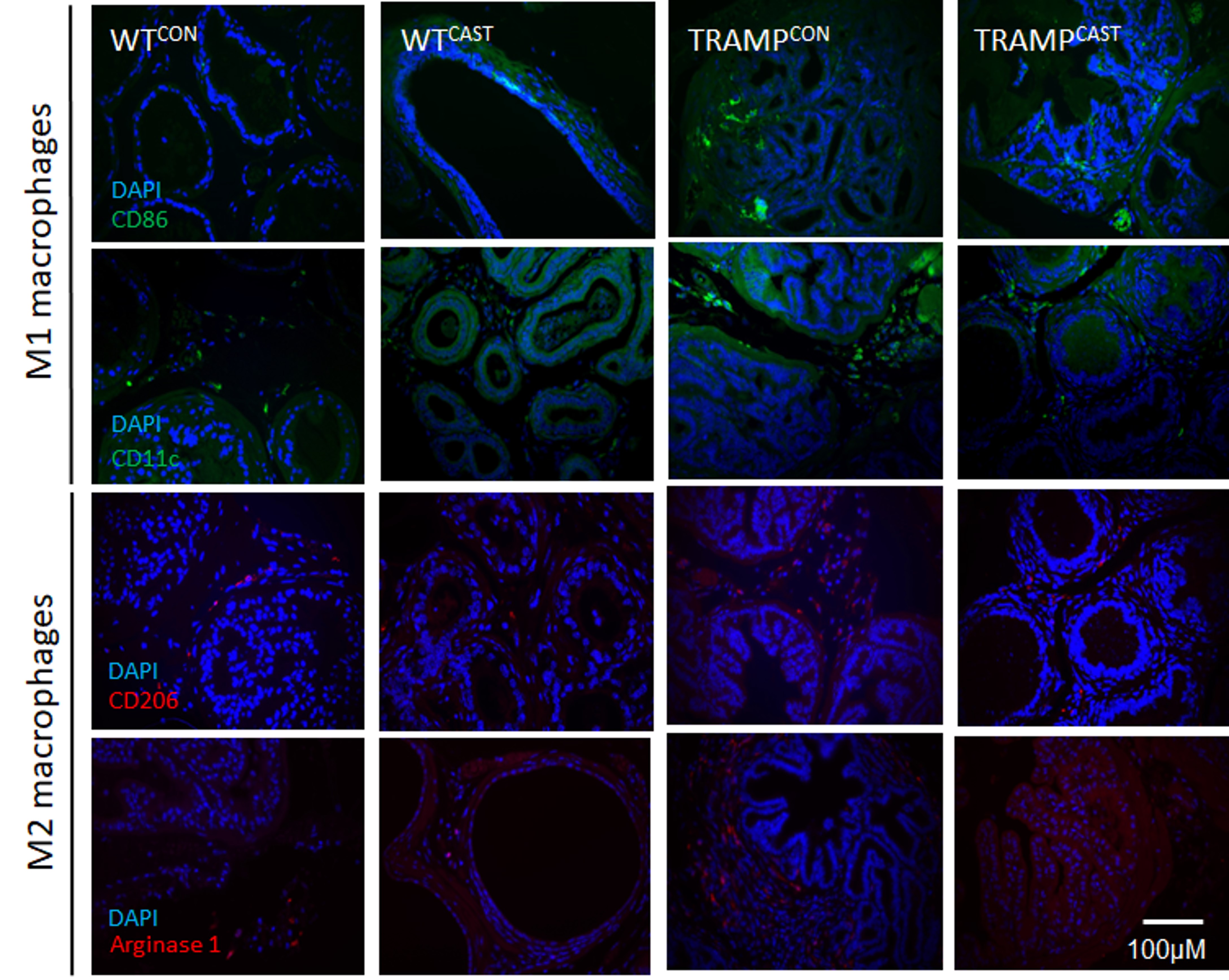

Supplement: Supplementary file 8 — Additional file 7:Figure S4. Strategies that produce brown/beige fat in the PPAT did not alter macrophage polarization. (A) Macrophage polarization in the prostate microenvironment of WT and TRAMP mice after castration for 12 weeks. CD86 and CD11c (M1 macrophage markers), CD206, arginase1 (M2 macrophage markers). Images were taken at 400X of magnification. [file 12964_2023_1294_MOESM7_ESM.jpg]
